# Crystal structure of a 1:1 co-crystal of quabodepistat (OPC-167832) with 2,5-di­hydroxy­benzoic acid using microcrystal electron diffraction

**DOI:** 10.1107/S2056989023006047

**Published:** 2023-09-19

**Authors:** Nasa Sakamoto, Katsuhiko Gato

**Affiliations:** a Preformulation Research Laboratory, CMC Headquarters, Otsuka Pharmaceutical Co., Ltd., Tokushima, 771-0182, Japan; Universidad de Los Andes Mérida, Venezuela

**Keywords:** crystal structure, co-crystal, quabodepistat, 2,5-di­hydroxy­benzoic acid, microcrystal electron diffraction

## Abstract

Quabodepistat [(5-{[(3*R*,4*R*)-1-(4-chloro-2,6-di­fluoro­phen­yl)-3,4-di­hydroxy­piperidin-4-yl]meth­oxy}-8-fluoro-3,4-di­hydro­quinolin-2(1*H*)-one); C_21_H_20_ClF_3_N_2_O_4_] and 2,5-di­hydroxy­benzoic acid (2,5DHBA; C_7_H_6_O_4_) were successfully co-crystallized. Given the small size of the crystals (1 × 0.2 × 0.2 µm) the structure was solved *via* microcrystal electron diffraction (MicroED). The C—O and C=O bond-length ratio of the carb­oxy­lic group in 2,5DHBA is 1.08 (1.34 Å/1.24 Å), suggesting that 2,5DHBA remains protonated. Therefore, the material is a co-crystal rather than a salt. The amide group of quabodepistat participates in a cyclic hydrogen bond with the carb­oxy­lic group of the 2,5DHBA. Additional hydrogen bonds involving the quabodepistat amide and hydroxyl groups result in a three-dimensional network.

## Abstract 2

A co-crystal of quabodepistat and 2,5-di­hydroxy­benzoic acid was obtained and the crystal structure was solved from microcrystal electron diffraction (MicroED) data.

## 1. Chemical context

Quabodepistat (OPC-167832), discovered by Otsuka Pharmaceutical Co., Ltd. as an anti-tuberculosis drug (Hariguchi *et al.*, 2020 ▸), has a mode of action that involves inhibiting the DprE1 enzyme of *M. tuberculosis*. 2,5-di­hydroxy­benzoic acid (2,5DHBA) – a derivative of benzoic acid or salicylic acid – is one of the hepatic metabolites of acetyl­salicylic acid (aspirin) (Levy & Tsuchiya, 1972 ▸). In the pharmaceutical industry, crystal-engineering approaches such as co-crystallization have been useful techniques for modifying the physicochemical properties [*e.g*., solubility (Yoshimura *et al.*, 2017 ▸) or tabletability (Wang *et al.*, 2021 ▸)] of an active pharmaceutical ingredient. We obtained the quabodepistat co-crystal with 2,5DHBA by the anti-solvent crystallization method and then attempted to solve its crystal structure using a conventional X-ray diffractometer; however, the crystal size was too small (1 × 0.2 × 0.2 µm). Therefore, we used MicroED (XtaLAB Synergy-ED, Rigaku Corporation, Tokyo, Japan), which is a powerful tool to solve crystal structures when the crystal size is smaller than 1 µm (Ito *et al.*, 2021 ▸). Here, we report the crystal structure of the 1:1 co-crystal between quabodepistat and 2,5DHBA, solved using MicroED. 
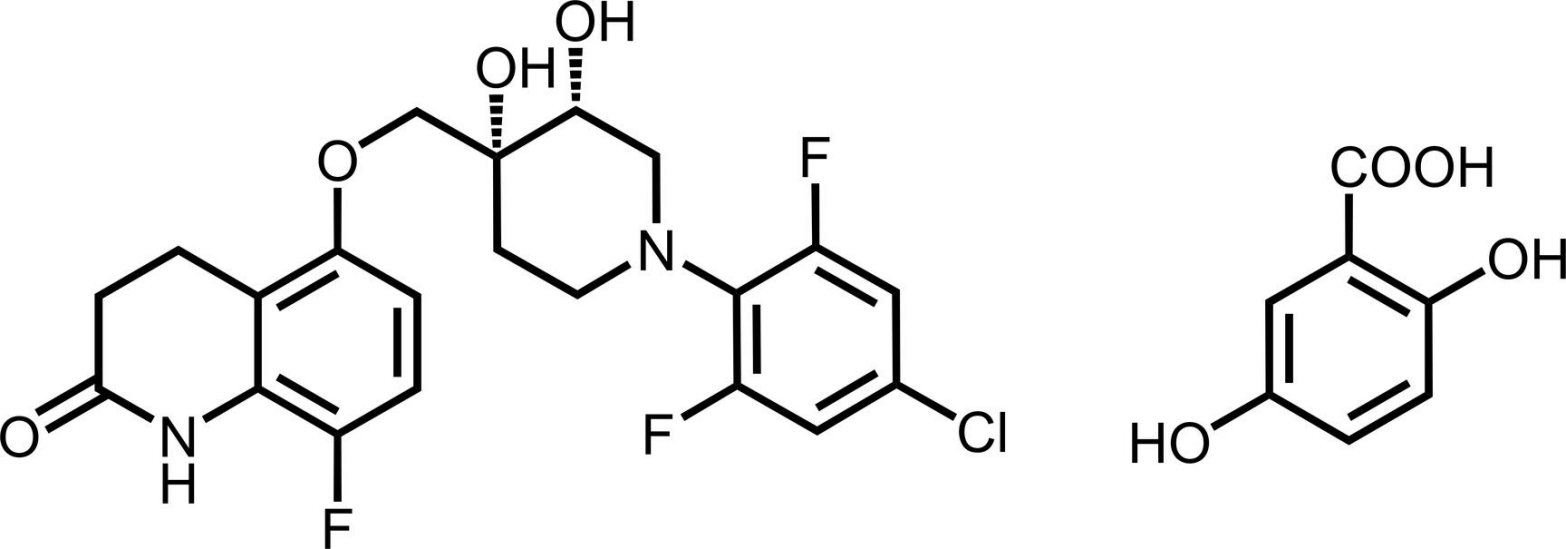


## 2. Structural commentary

Quabodepistat and 2,5DHBA co-crystallize in a 1:1 stoichiometric ratio in the monoclinic system, space group *P*2_1_, with *Z* = 2. Unusual bond lengths and angles are expected given the low crystal quality and the current limitations of the technique. A *Mogul* geometry analysis (Bruno *et al.*, 2004 ▸) indicated that the bonds C8—N7, O42—C36, F30—C28, C6—N7, O1—C2, and O41—C33, are unusual (*z*-score > 3). The angles O21—C16—C17, F30—C28—C23, C6—N7—C8, C9—C10—C11, O1—C14—C15, and C6—C11—C2 also have *z*-score values greater than 3.

All rings expected to be planar due to aromaticity (C2–C6/C11, C23–C28, and C32–C37) exhibit χ^2^ values (*PLATON*; Spek, 2020 ▸) indicating good planarity. The six-membered ring formed by C15–C17/N18/C19–C20 displays a slight chair conformation. The best plane constructed through atoms C23–C28 makes an angle of 20.9 (11)° with the best plane through C2–C6/N7/C8–C11.

## 3. Supra­molecular features

**Figure 1:**
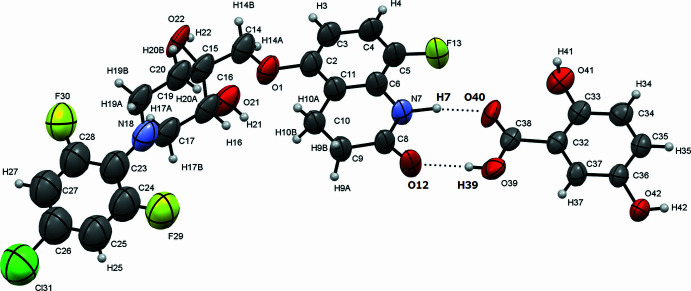
The mol­ecular structure of the quabodepistat:2,5DHBA co-crystal showing the carb­oxy­lic group and amide hydrogen bond synthon. Displacement ellipsoids are drawn at the 50% probability level.

**Figure 2:**
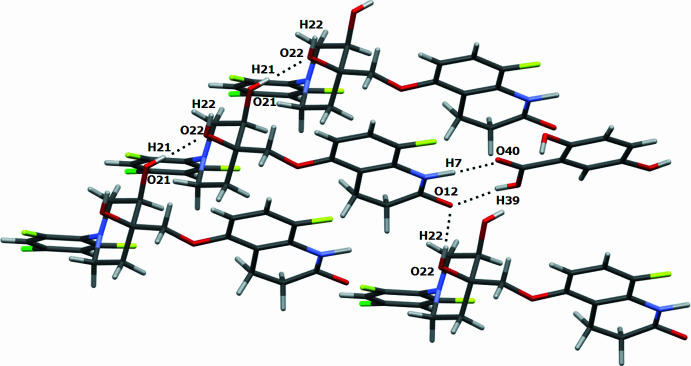
Inter­molecular inter­actions *via* hydrogen bonds in the quabodepistat:2,5DHBA co-crystal.

Inter­molecular inter­actions *via* hydrogen bonds are observed between quabodepistat and 2,5DHBA. One of the inter­actions is between a carb­oxy­lic group and an amide. As shown in Fig. 1 ▸, they form the common synthon: (amide of quabo­depistat) N7—H7⋯O40=C38 (carb­oxy­lic group of 2,5DHBA) and (amide of quabodepistat) C8=O12⋯H39—O39 (carb­oxy­lic group of 2,5DHBA). Moreover, the C8=O12 of the amide inter­acts with a hydroxyl group of a neighboring quabodepistat (O12⋯H22—O22), and the H22—O22 inter­acts with another hydroxyl of quabodepistat (O22⋯H21—O21). These inter­actions form a three-dimensional network (Figs. 2 ▸ and 3 ▸, Table 1 ▸). It is worth mentioning that the C—O:C=O bond-length ratio of the carb­oxy­lic group in 2,5DHBA is 1.08 (1.34 Å/1.24 Å), which suggests that protonation has not occurred for complex binding. Therefore, this material is a co-crystal instead of a salt. The compound TAK-020 has also been reported as a co-crystal with 2,5DHBA (Kimoto *et al.*, 2020 ▸). Therein, a carb­oxy­lic group of 2,5DHBA inter­acts with an amide moiety of the triazolinone of TAK-020, which is similar to the synthon observed in the compound reported in this contribution.

## 4. Database survey

A search for co-crystals with 2,5-di­hydroxy­benzoic acid (or gentisic acid) in the Cambridge Structural Database (WebCSD, accessed June 2023; Groom *et al.*, 2016 ▸) gave a total of 55 hits. In contrast, a search for co-crystals of quabodepistat with 2,5DHBA in the SciFinder database gave a total of two hits (Sakamoto & Miyata, 2021 ▸).

## 5. Synthesis and crystallization

Quabodepistat was synthesized at Otsuka Pharmaceutical Co., Ltd. (Tokushima, Japan). Tetra­hydro­furan (THF) and hexane were purchased from FUJIFILM Wako Pure Chemical Corporation (Osaka, Japan). 2,5DHBA was purchased from Tokyo Kasei Kogyo Co., Ltd. (Tokyo, Japan). Quabodepistat (5 g) and 2,5DHBA (16.9 g, stoichiometric ratio 1:10) were dissolved in 100 mL of THF. 250 mL of hexane were added while stirring. Precipitation occurred as soon as hexane was added. The THF/hexane was stirred at room temperature (approximately 298 K) for three days. After filtration, it was dried at room temperature for 24 h, then heated at 383 K for 20 h.

## 6. Refinement

Crystal data, data collection and structure refinement details are summarized in Table 2 ▸. Two data sets were merged to obtain 93.1% data completeness to 0.9 Å resolution. Crystals were illuminated at an electron dose rate of ∼0.01 e^−^Å^−2^ s^−1^. Contiguous diffraction frames were collected every 0.5° from each crystal by continuously rotating the sample stage at a goniometer rotation speed of 1° s^−1^; the sample stage was rotated from −40° to 40° for the first crystal (crystal 1) and from −60° to 60° for the second crystal (crystal 2). The structure was refined kinematically. Refinement with *SHELXL* was carried out using the scattering factors for electron diffraction (Saha *et al.*, 2022 ▸). Pseudo-merohedric twinning was identified and refined as described by Parkin (2021 ▸). For absolute structure determination, dynamical refinement is required. However, it was not performed since the absolute configuration of quabodepistat, which has two stereocenters, is known. Extinction was high because of the dynamical effects of electron diffraction (Saha *et al.*, 2022 ▸). In spite of the presence of some unusual bond lengths and angles, no unusual inter­molecular contacts are observed. This indicates that the structural model presented is correct.

## Supplementary Material

Crystal structure: contains datablock(s) global, I. DOI: 10.1107/S2056989023006047/dj2052sup1.cif

Supporting information file. DOI: 10.1107/S2056989023006047/dj2052Isup2.cml

Deposit the raw data into Zenodo: https://doi.org/10.5281/zenodo.7156704

Deposit the raw data into CCDC: https://dx.doi.org/10.5517/ccdc.csd.cc2d19zr

CCDC reference: 2205804

Additional supporting information:  crystallographic information; 3D view; checkCIF report

## Figures and Tables

**Figure 3:**
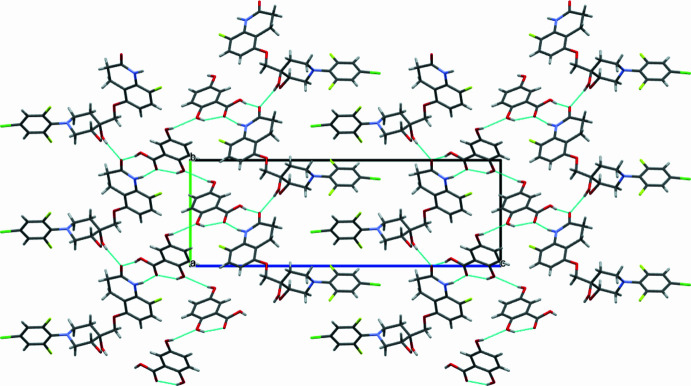
Crystal packing viewed down the *a* axis of the quabodepistat:2,5DHBA co-crystal.

**Table 1:** Hydrogen-bond geometry (Å, °)

*D*—H⋯*A*	*D*—H	H⋯*A*	*D*⋯*A*	*D*—H⋯*A*
C3—H3⋯O42^i^	0.93	2.71	3.40 (7)	131
C9—H9*B*⋯O22^ii^	0.97	2.94	3.49 (11)	117
N7—H7⋯O40^ii^	1.01	1.93	2.93 (9)	169
C34—H34⋯O42^iii^	0.93	2.69	3.48 (7)	143
O42—H42⋯O41^iv^	0.82	2.48	3.23 (6)	152
O39—H39⋯O12^v^	0.82	1.92	2.73 (10)	169
O21—H21⋯O22^vi^	0.82	2.02	2.84 (18)	175
O22—H22⋯O12^v^	0.82	2.11	2.90 (7)	164

Symmetry codes: (i) 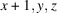 ; (ii) 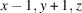 ; (iii) 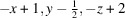 ; (iv) 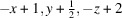 ; (v) 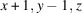 ; (vi) 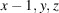 .

**Table 2:** Experimental details

Crystal data	
Chemical formula	C_21_H_20_ClF_3_N_2_O_4_·C_7_H_6_O_4_
*M*_r_	610.96
Crystal system, space group	Monoclinic, *P*2_1_
Temperature (K)	293
*a*, *b*, *c* (Å)	5.6 (3), 9.6 (3), 28.2 (3)
β (°)	90.30 (9)
*V* (Å^3^)	1516 (109)
*Z*	2
Radiation type	Electron, λ = 0.0251 Å
Crystal size (μm)	1.0 × 0.2 × 0.2

Data collection	
Diffractometer	Rigaku XtaLAB Synergy-ED
No. of measured, independent and observed [*I* > 2σ(*I*)] reflections	8548, 4096, 2030
*R*_int_	0.149
θ_max_ (°)	0.8
(sin θ/λ)_max_ (Å^−1^)	0.556

Refinement	
*R*[*F* ^2^ > 2σ(*F* ^2^)], *wR*(*F* ^2^), *S*	0.160, 0.480, 1.08
No. of reflections	4096
No. of parameters	348
No. of restraints	537
H-atom treatment	H-atom parameters constrained
Δρ_max_, Δρ_min_ (e Å^−3^)	0.15, −0.15

Computer programs: *CrysAlis PRO* (Rigaku OD, 2022 ▸), *SHELXD* (Sheldrick, 2008 ▸), *SHELXL2018/3* (Sheldrick, 2015 ▸) and *OLEX2* (Dolomanov *et al.*, 2009 ▸).

## supplementary crystallographic information

### Crystal data

**Table:**

C_21_H_20_ClF_3_N_2_O_4_·C_7_H_6_O_4_	*F*(000) = 224
*M**_r_* = 610.96	*D*_x_ = 1.338 Mg m^−^^3^
Monoclinic, *P*2_1_	Electron radiation, λ = 0.0251 Å
*a* = 5.6 (3) Å	Cell parameters from 411 reflections
*b* = 9.6 (3) Å	θ = 0.1–0.8°
*c* = 28.2 (3) Å	µ = 0.000 mm^−^^1^
β = 90.30 (9)°	*T* = 293 K
*V* = 1516 (109) Å^3^	Thin platelets, colourless
*Z* = 2	1 × 0.2 × 0.2 mm

### Data collection

**Table:**

Rigaku XtaLAB Synergy-ED diffractometer	2030 reflections with *I* > 2σ(*I*)
Radiation source: thermionic-emission electron gun	*R*_int_ = 0.149
Detector resolution: 10.0 pixels mm^-1^	θ_max_ = 0.8°, θ_min_ = 0.1°
rotation scans	*h* = −6→6
8548 measured reflections	*k* = −10→10
4096 independent reflections	*l* = −31→31

### Refinement

**Table:**

Refinement on *F*^2^	H-atom parameters constrained
Least-squares matrix: full	*w* = 1/[σ^2^(*F*_o_^2^) + (0.2805*P*)^2^ + 0.170*P*] where *P* = (*F*_o_^2^ + 2*F*_c_^2^)/3
*R*[*F*^2^ > 2σ(*F*^2^)] = 0.160	(Δ/σ)_max_ = 0.001
*wR*(*F*^2^) = 0.480	Δρ_max_ = 0.15 e Å^−^^3^
*S* = 1.08	Δρ_min_ = −0.15 e Å^−^^3^
4096 reflections	Extinction correction: '*SHELXL2018/3* (Sheldrick, 2015)', Fc^*^=kFc[1+0.001xFc^2^λ^3^/sin(2θ)]^-1/4^
348 parameters	Extinction coefficient: 368 (31)
537 restraints	Absolute structure: All f" are zero, so absolute structure could not be determined
Hydrogen site location: inferred from neighbouring sites	

### Special details

**Table:**

Geometry. All esds (except the esd in the dihedral angle between two l.s. planes) are estimated using the full covariance matrix. The cell esds are taken into account individually in the estimation of esds in distances, angles and torsion angles; correlations between esds in cell parameters are only used when they are defined by crystal symmetry. An approximate (isotropic) treatment of cell esds is used for estimating esds involving l.s. planes.
Refinement. Refined as a 2-component twin.

### Fractional atomic coordinates and isotropic or equivalent isotropic displacement parameters (Å^2^)

**Table:**

	*x*	*y*	*z*	*U*_iso_*/*U*_eq_	
C2	0.960 (3)	0.5589 (17)	0.7945 (6)	0.070 (5)	
C11	0.804 (4)	0.6700 (17)	0.7886 (5)	0.068 (4)	
C6	0.660 (3)	0.7121 (17)	0.8261 (6)	0.063 (4)	
C5	0.673 (3)	0.6431 (19)	0.8694 (6)	0.072 (5)	
C4	0.829 (3)	0.5320 (19)	0.8752 (5)	0.075 (5)	
H4	0.837583	0.485853	0.904220	0.090*	
C3	0.973 (3)	0.4899 (16)	0.8378 (6)	0.073 (5)	
H3	1.077324	0.415597	0.841680	0.087*	
C10	0.796 (5)	0.753 (2)	0.7416 (8)	0.069 (5)	
H10A	0.957975	0.785032	0.735893	0.083*	
H10B	0.759602	0.686397	0.716684	0.083*	
C9	0.631 (5)	0.878 (3)	0.7338 (10)	0.075 (5)	
H9A	0.528342	0.859999	0.706630	0.090*	
H9B	0.726751	0.959210	0.726908	0.090*	
C8	0.476 (5)	0.906 (3)	0.7778 (9)	0.068 (5)	
N7	0.479 (5)	0.824 (2)	0.8206 (10)	0.067 (5)	
H7	0.359249	0.842859	0.846624	0.080*	
C32	0.785 (2)	0.0552 (14)	0.9171 (6)	0.053 (4)	
C37	0.611 (3)	0.1500 (15)	0.9033 (5)	0.054 (4)	
H37	0.608891	0.184541	0.872468	0.065*	
C36	0.440 (2)	0.1933 (15)	0.9357 (6)	0.057 (5)	
C35	0.443 (2)	0.1417 (17)	0.9818 (6)	0.059 (5)	
H35	0.327933	0.170661	1.003412	0.071*	
C34	0.617 (3)	0.0469 (18)	0.9955 (5)	0.064 (5)	
H34	0.619200	0.012343	1.026363	0.076*	
C33	0.789 (2)	0.0036 (15)	0.9632 (6)	0.059 (4)	
O40	1.128 (4)	−0.085 (2)	0.8909 (11)	0.069 (6)	
C15	1.307 (4)	0.340 (2)	0.7081 (11)	0.081 (5)	
C38	0.975 (3)	0.005 (2)	0.8818 (8)	0.053 (4)	
O42	0.251 (4)	0.291 (2)	0.9211 (10)	0.061 (6)	
H42	0.156468	0.302044	0.943037	0.092*	
C14	1.241 (6)	0.397 (3)	0.7565 (12)	0.082 (5)	
H14A	1.151605	0.326933	0.773690	0.099*	
H14B	1.386475	0.415660	0.774296	0.099*	
O12	0.319 (5)	1.000 (2)	0.7774 (9)	0.075 (7)	
O41	0.958 (4)	−0.099 (2)	0.9792 (11)	0.076 (7)	
H41	1.043887	−0.121798	0.956939	0.114*	
F13	0.511 (6)	0.688 (2)	0.9048 (10)	0.079 (7)	
O1	1.104 (4)	0.520 (2)	0.7540 (12)	0.077 (5)	
O39	0.968 (4)	0.071 (2)	0.8400 (11)	0.066 (5)	
H39	1.072100	0.039139	0.822607	0.099*	
C17	1.138 (5)	0.243 (2)	0.6290 (10)	0.090 (6)	
H17A	1.232176	0.158593	0.628400	0.108*	
H17B	0.988301	0.225125	0.612765	0.108*	
O21	0.972 (5)	0.185 (3)	0.7110 (12)	0.098 (8)	
H21	0.828359	0.200591	0.711408	0.148*	
C20	1.438 (5)	0.447 (2)	0.6775 (10)	0.089 (6)	
H20A	1.346328	0.532911	0.678027	0.106*	
H20B	1.591213	0.466927	0.692442	0.106*	
O22	1.469 (3)	0.224 (2)	0.7149 (14)	0.083 (7)	
H22	1.401436	0.161832	0.729667	0.125*	
C16	1.087 (4)	0.283 (2)	0.6810 (9)	0.083 (5)	
H16	0.976517	0.361826	0.679004	0.100*	
C23	1.265 (6)	0.344 (3)	0.5511 (9)	0.131 (7)	
C28	1.434 (5)	0.264 (3)	0.5280 (10)	0.150 (8)	
C27	1.440 (5)	0.261 (3)	0.4787 (10)	0.164 (9)	
H27	1.553704	0.206657	0.463185	0.197*	
C26	1.277 (6)	0.339 (4)	0.4525 (9)	0.170 (9)	
C25	1.108 (5)	0.419 (3)	0.4756 (10)	0.162 (9)	
H25	0.998639	0.471406	0.458099	0.194*	
C24	1.102 (5)	0.422 (3)	0.5249 (10)	0.148 (8)	
Cl31	1.268 (6)	0.322 (4)	0.3905 (9)	0.215 (12)	
F29	0.929 (10)	0.501 (5)	0.548 (2)	0.172 (14)	
F30	1.615 (9)	0.176 (5)	0.551 (2)	0.158 (13)	
C19	1.485 (4)	0.410 (3)	0.6254 (10)	0.092 (6)	
H19A	1.534890	0.492210	0.608241	0.111*	
H19B	1.612402	0.341921	0.623696	0.111*	
N18	1.268 (5)	0.353 (3)	0.6034 (11)	0.101 (5)	

### Atomic displacement parameters (Å^2^)

**Table:**

	*U*^11^	*U*^22^	*U*^33^	*U*^12^	*U*^13^	*U*^23^
C2	0.078 (9)	0.057 (8)	0.076 (10)	0.029 (7)	0.010 (9)	0.005 (8)
C11	0.085 (9)	0.056 (8)	0.062 (9)	0.030 (7)	0.008 (8)	0.011 (7)
C6	0.087 (9)	0.043 (7)	0.059 (9)	0.034 (7)	0.009 (8)	0.015 (7)
C5	0.088 (10)	0.063 (9)	0.066 (10)	0.037 (8)	0.007 (9)	0.024 (8)
C4	0.081 (11)	0.069 (10)	0.074 (11)	0.037 (8)	0.002 (10)	0.020 (9)
C3	0.082 (11)	0.059 (9)	0.076 (11)	0.032 (9)	0.002 (10)	0.008 (8)
C10	0.089 (10)	0.058 (9)	0.060 (10)	0.031 (8)	0.009 (10)	0.011 (8)
C9	0.096 (11)	0.067 (9)	0.062 (11)	0.040 (9)	0.015 (10)	0.018 (9)
C8	0.090 (10)	0.058 (8)	0.055 (10)	0.045 (8)	0.011 (9)	0.013 (8)
N7	0.096 (10)	0.045 (8)	0.059 (10)	0.040 (8)	0.015 (9)	0.009 (7)
C32	0.051 (7)	0.050 (7)	0.057 (9)	0.005 (6)	0.002 (7)	−0.002 (7)
C37	0.055 (8)	0.052 (8)	0.055 (10)	0.010 (7)	0.008 (8)	−0.004 (8)
C36	0.049 (8)	0.064 (9)	0.059 (10)	0.015 (7)	0.004 (8)	−0.004 (8)
C35	0.052 (9)	0.063 (9)	0.062 (10)	0.001 (7)	0.009 (9)	0.003 (9)
C34	0.064 (9)	0.063 (9)	0.064 (10)	0.000 (8)	0.004 (9)	0.012 (9)
C33	0.061 (8)	0.053 (8)	0.065 (10)	−0.004 (7)	0.006 (8)	0.009 (8)
O40	0.047 (10)	0.078 (12)	0.083 (17)	0.016 (9)	0.025 (12)	0.016 (12)
C15	0.062 (8)	0.062 (8)	0.118 (11)	0.038 (7)	0.019 (9)	0.020 (8)
C38	0.044 (8)	0.053 (9)	0.063 (10)	0.001 (7)	0.001 (8)	−0.008 (8)
O42	0.048 (10)	0.065 (11)	0.072 (16)	0.022 (8)	0.004 (12)	−0.005 (11)
C14	0.072 (10)	0.059 (9)	0.116 (12)	0.033 (9)	0.018 (10)	0.011 (9)
O12	0.106 (15)	0.062 (11)	0.058 (15)	0.059 (11)	0.015 (13)	0.020 (11)
O41	0.087 (14)	0.055 (10)	0.087 (17)	0.001 (9)	0.013 (14)	0.024 (12)
F13	0.134 (17)	0.045 (9)	0.059 (14)	0.041 (11)	0.023 (13)	0.025 (10)
O1	0.071 (9)	0.057 (8)	0.103 (12)	0.030 (8)	0.018 (10)	0.007 (9)
O39	0.046 (10)	0.084 (12)	0.069 (13)	0.001 (10)	0.000 (11)	0.005 (11)
C17	0.075 (10)	0.072 (10)	0.124 (13)	0.041 (9)	0.019 (11)	0.018 (10)
O21	0.068 (12)	0.092 (14)	0.14 (2)	0.016 (10)	0.013 (15)	0.031 (14)
C20	0.074 (10)	0.067 (9)	0.125 (13)	0.034 (8)	0.020 (11)	0.026 (10)
O22	0.049 (10)	0.057 (10)	0.14 (2)	0.036 (9)	0.027 (13)	0.012 (12)
C16	0.062 (9)	0.065 (9)	0.122 (12)	0.039 (8)	0.019 (10)	0.018 (9)
C23	0.123 (13)	0.128 (12)	0.143 (14)	0.041 (12)	0.019 (14)	0.012 (13)
C28	0.146 (15)	0.149 (15)	0.154 (16)	0.043 (14)	0.018 (16)	−0.007 (15)
C27	0.165 (16)	0.162 (16)	0.166 (18)	0.041 (15)	0.017 (18)	−0.011 (17)
C26	0.174 (16)	0.168 (16)	0.168 (17)	0.040 (15)	0.015 (18)	−0.007 (16)
C25	0.161 (16)	0.159 (16)	0.165 (18)	0.040 (15)	0.015 (18)	0.005 (17)
C24	0.144 (15)	0.146 (15)	0.153 (17)	0.045 (14)	0.018 (16)	0.012 (15)
Cl31	0.23 (2)	0.22 (2)	0.19 (2)	0.06 (2)	0.01 (2)	−0.01 (2)
F29	0.17 (3)	0.18 (3)	0.16 (3)	0.05 (2)	0.02 (3)	0.02 (2)
F30	0.16 (2)	0.14 (2)	0.17 (3)	0.05 (2)	0.01 (3)	−0.05 (2)
C19	0.078 (11)	0.072 (10)	0.127 (13)	0.041 (9)	0.025 (12)	0.030 (11)
N18	0.088 (10)	0.090 (10)	0.126 (12)	0.035 (9)	0.022 (11)	0.023 (10)

### Geometric parameters (Å, º)

**Table:**

C2—C11	1.3900	C15—O22	1.45 (5)
C2—C3	1.3900	C15—C16	1.54 (6)
C2—O1	1.45 (4)	C38—O39	1.34 (4)
C11—C6	1.3900	O42—H42	0.8200
C11—C10	1.55 (2)	C14—H14A	0.9700
C6—C5	1.3900	C14—H14B	0.9700
C6—N7	1.49 (6)	C14—O1	1.41 (5)
C5—C4	1.3900	O41—H41	0.8200
C5—F13	1.42 (5)	O39—H39	0.8200
C4—H4	0.9300	C17—H17A	0.9700
C4—C3	1.3900	C17—H17B	0.9700
C3—H3	0.9300	C17—C16	1.54 (2)
C10—H10A	0.9700	C17—N18	1.47 (4)
C10—H10B	0.9700	O21—H21	0.8200
C10—C9	1.53 (5)	O21—C16	1.42 (3)
C9—H9A	0.9700	C20—H20A	0.9700
C9—H9B	0.9700	C20—H20B	0.9700
C9—C8	1.54 (4)	C20—C19	1.53 (2)
C8—N7	1.44 (4)	O22—H22	0.8200
C8—O12	1.26 (5)	C16—H16	0.9800
N7—H7	1.0100	C23—C28	1.3900
C32—C37	1.3900	C23—C24	1.3900
C32—C33	1.3900	C23—N18	1.47 (4)
C32—C38	1.53 (5)	C28—C27	1.3900
C37—H37	0.9300	C28—F30	1.46 (7)
C37—C36	1.3900	C27—H27	0.9300
C36—C35	1.3900	C27—C26	1.3900
C36—O42	1.47 (6)	C26—C25	1.3900
C35—H35	0.9300	C26—Cl31	1.75 (3)
C35—C34	1.3900	C25—H25	0.9300
C34—H34	0.9300	C25—C24	1.3900
C34—C33	1.3900	C24—F29	1.40 (7)
C33—O41	1.44 (5)	C19—H19A	0.9700
O40—C38	1.24 (5)	C19—H19B	0.9700
C15—C14	1.52 (4)	C19—N18	1.46 (6)
C15—C20	1.54 (4)		
			
C11—C2—C3	120.0	O40—C38—C32	124 (3)
C11—C2—O1	117 (2)	O40—C38—O39	122 (2)
C3—C2—O1	123 (2)	O39—C38—C32	114 (2)
C2—C11—C10	120.8 (16)	C36—O42—H42	109.5
C6—C11—C2	120.0	C15—C14—H14A	108.9
C6—C11—C10	119.2 (19)	C15—C14—H14B	108.9
C11—C6—N7	122 (2)	H14A—C14—H14B	107.7
C5—C6—C11	120.0	O1—C14—C15	113 (3)
C5—C6—N7	117.9 (18)	O1—C14—H14A	108.9
C6—C5—C4	120.0	O1—C14—H14B	108.9
C6—C5—F13	116 (2)	C33—O41—H41	109.5
C4—C5—F13	124 (2)	C14—O1—C2	119 (3)
C5—C4—H4	120.0	C38—O39—H39	109.5
C3—C4—C5	120.0	H17A—C17—H17B	107.9
C3—C4—H4	120.0	C16—C17—H17A	109.1
C2—C3—H3	120.0	C16—C17—H17B	109.1
C4—C3—C2	120.0	N18—C17—H17A	109.1
C4—C3—H3	120.0	N18—C17—H17B	109.1
C11—C10—H10A	106.6	N18—C17—C16	112 (2)
C11—C10—H10B	106.6	C16—O21—H21	109.5
H10A—C10—H10B	106.6	C15—C20—H20A	107.9
C9—C10—C11	123 (2)	C15—C20—H20B	107.9
C9—C10—H10A	106.6	H20A—C20—H20B	107.2
C9—C10—H10B	106.6	C19—C20—C15	117 (2)
C10—C9—H9A	109.3	C19—C20—H20A	107.9
C10—C9—H9B	109.3	C19—C20—H20B	107.9
C10—C9—C8	111 (3)	C15—O22—H22	109.5
H9A—C9—H9B	108.0	C15—C16—H16	105.0
C8—C9—H9A	109.3	C17—C16—C15	114 (3)
C8—C9—H9B	109.3	C17—C16—H16	105.0
N7—C8—C9	125 (2)	O21—C16—C15	107 (3)
O12—C8—C9	121 (3)	O21—C16—C17	119 (3)
O12—C8—N7	114 (3)	O21—C16—H16	105.0
C6—N7—H7	120.4	C28—C23—C24	120.0
C8—N7—C6	119 (3)	C28—C23—N18	120 (2)
C8—N7—H7	120.4	C24—C23—N18	120 (2)
C37—C32—C33	120.0	C23—C28—C27	120.0
C37—C32—C38	121 (2)	C23—C28—F30	126 (3)
C33—C32—C38	119.1 (19)	C27—C28—F30	114 (3)
C32—C37—H37	120.0	C28—C27—H27	120.0
C36—C37—C32	120.0	C26—C27—C28	120.0
C36—C37—H37	120.0	C26—C27—H27	120.0
C37—C36—C35	120.0	C27—C26—Cl31	119.7 (11)
C37—C36—O42	120 (3)	C25—C26—C27	120.0
C35—C36—O42	119.6 (18)	C25—C26—Cl31	120.1 (13)
C36—C35—H35	120.0	C26—C25—H25	120.0
C34—C35—C36	120.0	C26—C25—C24	120.0
C34—C35—H35	120.0	C24—C25—H25	120.0
C35—C34—H34	120.0	C23—C24—F29	120 (4)
C35—C34—C33	120.0	C25—C24—C23	120.0
C33—C34—H34	120.0	C25—C24—F29	120 (3)
C32—C33—O41	122.8 (19)	C20—C19—H19A	109.6
C34—C33—C32	120.0	C20—C19—H19B	109.6
C34—C33—O41	117 (3)	H19A—C19—H19B	108.1
C14—C15—C20	112 (3)	N18—C19—C20	110 (3)
C14—C15—C16	112 (3)	N18—C19—H19A	109.6
C20—C15—C16	110 (3)	N18—C19—H19B	109.6
O22—C15—C14	109 (3)	C17—N18—C23	116 (3)
O22—C15—C20	107 (4)	C19—N18—C17	118 (3)
O22—C15—C16	107 (4)	C19—N18—C23	117 (3)
			
C2—C11—C6—C5	0.0	C14—C15—C16—C17	172 (2)
C2—C11—C6—N7	−176 (2)	C14—C15—C16—O21	−54 (3)
C2—C11—C10—C9	−179 (2)	O12—C8—N7—C6	−178 (2)
C11—C2—C3—C4	0.0	F13—C5—C4—C3	176 (2)
C11—C2—O1—C14	−172 (2)	O1—C2—C11—C6	179.7 (19)
C11—C6—C5—C4	0.0	O1—C2—C11—C10	−3 (2)
C11—C6—C5—F13	−177 (2)	O1—C2—C3—C4	−180 (2)
C11—C6—N7—C8	−10 (3)	C20—C15—C14—O1	56 (4)
C11—C10—C9—C8	−1 (4)	C20—C15—C16—C17	46 (3)
C6—C11—C10—C9	−2 (4)	C20—C15—C16—O21	−180 (2)
C6—C5—C4—C3	0.0	C20—C19—N18—C17	−48 (3)
C5—C6—N7—C8	174 (2)	C20—C19—N18—C23	165 (2)
C5—C4—C3—C2	0.0	O22—C15—C14—O1	174 (2)
C3—C2—C11—C6	0.0	O22—C15—C20—C19	69 (3)
C3—C2—C11—C10	177 (2)	O22—C15—C16—C17	−70 (3)
C3—C2—O1—C14	8 (3)	O22—C15—C16—O21	65 (3)
C10—C11—C6—C5	−177 (2)	C16—C15—C14—O1	−69 (4)
C10—C11—C6—N7	7 (2)	C16—C15—C20—C19	−46 (3)
C10—C9—C8—N7	−2 (4)	C16—C17—N18—C23	−163 (3)
C10—C9—C8—O12	−176 (3)	C16—C17—N18—C19	51 (4)
C9—C8—N7—C6	7 (5)	C23—C28—C27—C26	0.0
N7—C6—C5—C4	176 (2)	C28—C23—C24—C25	0.0
N7—C6—C5—F13	−1 (2)	C28—C23—C24—F29	178.7 (11)
C32—C37—C36—C35	0.0	C28—C23—N18—C17	−86 (5)
C32—C37—C36—O42	−178.6 (16)	C28—C23—N18—C19	61 (5)
C37—C32—C33—C34	0.0	C28—C27—C26—C25	0.0
C37—C32—C33—O41	177.5 (17)	C28—C27—C26—Cl31	−174 (3)
C37—C32—C38—O40	−175.6 (19)	C27—C26—C25—C24	0.0
C37—C32—C38—O39	6 (2)	C26—C25—C24—C23	0.0
C37—C36—C35—C34	0.0	C26—C25—C24—F29	−178.7 (11)
C36—C35—C34—C33	0.0	C24—C23—C28—C27	0.0
C35—C34—C33—C32	0.0	C24—C23—C28—F30	−179 (3)
C35—C34—C33—O41	−177.7 (17)	C24—C23—N18—C17	97 (5)
C33—C32—C37—C36	0.0	C24—C23—N18—C19	−116 (4)
C33—C32—C38—O40	4 (3)	Cl31—C26—C25—C24	174 (3)
C33—C32—C38—O39	−174.3 (16)	F30—C28—C27—C26	179 (3)
C15—C14—O1—C2	159 (2)	N18—C17—C16—C15	−49 (3)
C15—C20—C19—N18	47 (3)	N18—C17—C16—O21	−177 (2)
C38—C32—C37—C36	179.5 (14)	N18—C23—C28—C27	−177 (3)
C38—C32—C33—C34	−179.5 (14)	N18—C23—C28—F30	4 (3)
C38—C32—C33—O41	−2 (2)	N18—C23—C24—C25	177 (3)
O42—C36—C35—C34	178.6 (16)	N18—C23—C24—F29	−5 (3)
C14—C15—C20—C19	−172 (2)		

### Hydrogen-bond geometry (Å, º)

**Table:**

*D*—H···*A*	*D*—H	H···*A*	*D*···*A*	*D*—H···*A*
C3—H3···O42^i^	0.93	2.71	3.40 (7)	131
C9—H9*B*···O22^ii^	0.97	2.94	3.49 (11)	117
N7—H7···O40^ii^	1.01	1.93	2.93 (9)	169
C34—H34···O42^iii^	0.93	2.69	3.48 (7)	143
O42—H42···O41^iv^	0.82	2.48	3.23 (6)	152
O39—H39···O12^v^	0.82	1.92	2.73 (10)	169
O21—H21···O22^vi^	0.82	2.02	2.84 (18)	175
O22—H22···O12^v^	0.82	2.11	2.90 (7)	164

Symmetry codes: (i) *x*+1, *y*, *z*; (ii) *x*−1, *y*+1, *z*; (iii) −*x*+1, *y*−1/2, −*z*+2; (iv) −*x*+1, *y*+1/2, −*z*+2; (v) *x*+1, *y*−1, *z*; (vi) *x*−1, *y*, *z*.
